# Best Practices in Recruitment and Outreach to Women and Diverse Veterans for Coronavirus Research at the U.S. Department of Veterans Affairs

**DOI:** 10.1089/heq.2023.0013

**Published:** 2023-05-26

**Authors:** Mary M. Klote, Claudia G. Gutierrez, Jennifer E. Deen, Lori L. Churby, Patrick Bateman, Victoria J. Davey

**Affiliations:** Office of Research and Development, Veterans Health Administration, Washington, District of Columbia, USA.

**Keywords:** Veterans, recruitment, COVID-19, coronavirus, research

## Abstract

In September 2020, the Department of Veterans Affairs (VA) launched a novel volunteer research registry to rapidly recruit eligible study participants for research on SARS-CoV-2 and COVID-19 vaccines and treatments at VA Medical Centers selected as study sites for COVID-19 clinical trials. Targeted multimedia outreach campaigns were used to recruit diverse populations, including those historically under-represented in medical research. By November 2022, 58,561 volunteers were enrolled in the registry, 19% of whom were women, 9% Hispanic/Latino, and 8% Black. The registry's strategic approach to outreach proved successful in recruiting diverse volunteers, with geotargeted e-mails recruiting the most diversity.

## Introduction

In September 2020, the U.S. Department of Veterans Affairs (VA) launched a volunteer registry on VA.gov known as the Coronavirus Research Volunteer List (CRVL) with the goal of improving the diversity and availability of eligible research participants to support VA research on SARS-CoV-2 and COVID-19 vaccines and treatments. Many of these studies were part of U.S. government-led public–private partnerships.

Veterans and all U.S. citizens aged 18 or older were eligible to sign-up. The highest recruitment priority was Black and Hispanic populations, as well as women and other racial and ethnic minorities who experienced disproportionate health impacts from COVID-19.^1,2^ These populations are historically under-represented in research and are among the most difficult to engage in clinical trials, especially COVID-19 studies.^3,4^

Until as late as 1989, the National Institutes of Health did not formally encourage the recruitment of women and minorities into research studies.^5^ Other reasons for low participation included a lack of trust in government and pharmaceutical research, as well as the health care system at large, especially among minority populations who historically experienced unethical medical research in private and government settings.^6,7^ Before the pandemic, Black Americans accounted for ∼14% of all clinical trial participants, and they continue to be under-represented in COVID-19 research.^8,9^

In addition, before the pandemic, many Americans held deep-rooted misconceptions about vaccines and were skeptical of COVID-19 vaccine clinical trials, believing the accelerated rollout compromised scientific rigor and safety protocols.^10–12^ Polling in 2020 found that Black Americans, Hispanics, and young adults were least likely to want a COVID-19 vaccine.^13^

The volunteer list implemented a targeted recruitment campaign to overcome these challenges and encourage diverse participation in COVID-19 vaccine clinical trials representative of the American public and those populations most impacted by the disease.

## Materials and Methods

In October 2020, CRVL launched two parallel outreach and recruitment campaigns: a nationwide campaign and local campaigns at VA Medical Centers (VAMCs) selected as study sites for clinical trials for COVID-19 vaccine candidates and treatments.

Both local and national campaigns involved a multichannel approach using print and digital media, including a communication toolkit, geo-targeted mailings and e-mail campaigns, proactive and reactive media engagement, social media, human-interest stories posted to VA's main news site, and feature stories posted to VAMC websites.

The communication toolkit consisted of a one-pager, two outreach flyers, a flyer designed to solicit support from VA employees, a social media toolkit for VAMCs, a PowerPoint slide presentation for outreach to frontline VA staff, approved core messages for media requests, digital billboards for VAMCs, and text to be included in patient instructions to assist with referrals to the registry (Supplementary Files S1 and S2).

All toolkit materials included images of people of color, reflecting the campaign's primary target audience. Spanish translations were later made available to better reach Hispanic populations. All materials were approved by VA's COVID-19 Joint Task Force and the Institutional Review Board that oversees CRVL. In addition, a Diversity and Inclusion Subcommittee within the VA National Research Advisory Council was established to ensure CRVL messaging, materials, and tactics were culturally sensitive and effective.

Messaging focused on COVID-19 safety and prevention for the individual as a way to protect family members and loved ones from illness, and dispelling misconceptions about vaccines in general, and COVID-19 vaccines in particular.^13,14^ CRVL materials featured images of family, highlighted the stories of diverse Veteran volunteers who were also parents and grandparents, and reassured participants that they would *not* be deliberately infected with COVID-19 as part of any study.

The national campaign included an episode on VA's weekly podcast, Borne the Battle, and a VA press release that announced national recruitment efforts for COVID-19 research volunteers for, triggering coverage in media outlets across the country.^15,16^ A 30-sec public service announcement (PSA) was produced that featured Anthony Fauci, MD, Director of the National Institute of Allergy and Infectious Disease, Jerome Adams, MD, the 20th Surgeon General of the United States and a Black Veteran, and Michele S. Jones, a Black female Veteran who was the highest-ranking enlisted African American in the Army Reserve.^17,18^ Human-interest stories on diverse Veteran volunteers were published to VA's main news site and shared across VA social media.^19–24^

Local campaigns included geotargeted mass e-mails and mailings through VA's Veteran Experience Office and VA's Million Veteran Program to inform Veterans of COVID-19 vaccine trials at their nearby VA, and the opportunity to participate by signing up for CRVL. Feature stories were also published on the websites of VAMCs participating in vaccine clinical trials, with information on vaccine trials at each VA facility, and instructions on enrollment for CRVL; these stories were e-mailed to all subscribers of each facility.

## Results

In <75 days, the CRVL campaign recruited >50,000 volunteers. By November 2022, the list grew to 58,561 volunteers, 19% of whom are women, 9% Hispanic/Latino, and 8% Black (Table 1).

**Table 1.** Coronavirus Research Volunteer List Volunteer Demographics by Sex, Gender, Race, and Ethnicity as of November 2022

	Total	%
Total No. of volunteers	58,561	
Sex and gender		
Male	47,296	81%
Female	10,995	19%
Transgender female	99	0%
Transgender male	50	0%
Nonbinary	104	0%
Self-identify	29	0%
None of the above	58	0%
Race and ethnicity		
American Indian Alaska Native	1621	3%
Asian	1438	2%
Black African American	4540	8%
Hispanic Latino Spanish Origin	5454	9%
Hawaiian Pacific Islander	374	1%
White	47,436	81%
Other Race Ethnicity	657	1%
None of the above	593	1%

CRVL supported recruitment for four national COVID-19 vaccine clinical trials at 20 VAMCs and the Researching COVID-19 to Enhance Recovery (RECOVER) Initiative, a large-scale study on the long-term effects of COVID-19. As of March 2022, information on 3574 potential volunteers was provided to study teams, matching age, ethnicity, and minority enrollment criteria. Of these volunteers, 1764 were contacted by the study teams and 437 were enrolled (Table 2).

**Table 2.** Known Coronavirus Research Volunteer List Volunteers Provided to Veterans Affairs COVID-19 Trials

Trial	Total volunteers provided	Women		Minority		Total No. contacted by study teams		Total No. contacted and enrolled	
		Count	%^a^	Count	%^a^	Count	%^a^	Count	%^b^
Janssen (16 sites)	2813	699	25%	1454	52%	1373	49%	325	24%
Moderna (1 site_	36	7	19%	6	17%	23	64%	11	48%
AstraZeneca (1 site)	344	77	22%	176	51%	96	28%	31	32%
Novavax (2 sites)	280	77	28%	226	81%	212	76%	47	22%
RECOVER (1 site)	101	16	16%	18	18%	60	59%	23	38%
Total volunteers provided	3574	876	25%	1880	53%	1764	50%	437	25%

The number of recruits into trials for registry volunteers with COVID-19 were not able to be counted.

^a^ % is the percentage of total CRVL volunteers provided to study teams for each study.

^b^ % of CRVL volunteers provided to and contacted by study teams who were successfully enrolled into clinical trials.

CRVL, Coronavirus Research Volunteer List; RECOVER, Researching COVID-19 to Enhance Recovery.

Success of national campaign efforts were tracked based on the number of new volunteer sign-ups, including the number of diverse volunteers, within 24, 48, and 78 h after each activity (Table 3).

**Table 3.** Most Successful Campaign Activities by Number of New Volunteer Sign-Ups

Date	Campaign tactic	Total distribution	Geographical area	No. of new volunteers within 24 h		No. of new volunteers within 48 h		No. of new volunteers within 72 h	
				Non-White	Total	Non-White	Total	Non-White	Total
November 16, 2020	Janssen Feature Story^a^	E-mail sent to GovDelivery subscribers of Columbia VAMC	Columbia, SC		104		200	27	262
November 16, 2020	National press release	Published on va.gov	Nationwide	282	1434	422	2089	3341	16,238
November 16, 2020	ORD newsletter spotlight	∼92,000	Nationwide	282	1434	422	2089	3341	16,238
November 18, 2020	MyHealtheVet newsletterspotlight	2,000,000+ Veteran subscribers	Nationwide	527	3355	3425	21,595	3809	23,589
November 18, 2020	VetResources newsletter spotlight	12,000,000+ GovDelivery e-mail subscribers	Nationwide	527	3355	3475	21,594	3809	23,589
November 23, 2020	Tweet (PSA)	109,100 followers of @veteranshealth	Nationwide	742	2468	1135	3653	2054	7575
November 23, 2020	Geo-targeted e-mail for Janssen vaccine clinical sites	214,000 e-mail recipients	Catchment area of 16 VAMCs with Janssen trial	742	2468	1135	3653	2054	7575
November 24, 2020	VAntage Point blog	201 views	Nationwide	393	1185	1312	5107	2017	8781

^a^ No. of new non-White volunteers could not be tracked within 24 and 48 h after the Janssen feature story published to the Columbia VA website.

PSA, public service announcement; VA, Veterans Affairs; VAMCs, VA Medical Centers.

CRVL was featured in VA's largest weekly e-mail newsletter, sent to >12 million GovDelivery e-mail subscribers, and also featured in the My HealtheVet's weekly e-mail newsletter to >2 million Veteran subscribers. Together, both tactics resulted in 21,594 new volunteers in 48 h, including 3475 volunteers of diverse racial/ethnic backgrounds.

Use of geotargeted e-mails to >214,000 Veterans who were participating in the Janssen vaccine trial and lived within the catchment area of 16 VAMCs also proved to be successful. Within 72 h, 7575 new volunteers joined CRVL. Of these, 2054 (27%) were of diverse racial/ethnic backgrounds.

## Discussion

CRVL was established to connect Veterans and other Americans interested in COVID-19 research with studies in their area, thereby assisting researchers with recruitment needs. CRVL tailored messaging to focus on under-represented populations in research, such as minorities and women, to ensure a sufficient number of volunteers could meet the inclusions/exclusion criteria for COVID-19 trials.

By March 2022, 25% of selected CRVL volunteers were enrolled into research. Inclusion criteria for COVID-19 vaccine trials were dynamic and researchers were expected to adapt quickly to changes in enrollment criteria. As a result, some volunteers no longer met eligibility requirements. However, CRVL was able to continuously meet enrollment needs due to the large number of interested and diverse volunteers in the registry.

Campaign monitoring and evaluation was possible only for digital media content, as recruitment from print materials was largely untraceable. In addition, the most successful CRVL campaign activities occurred on or around the same day as other key activities. Upon close examination of overlapping communication tactics, the Vet Resources e-mail newsletter and geotargeted e-mail invitations appear to be responsible for recruiting the most volunteers and achieving the most diversity for CRVL.

In addition, we could not document CRVL volunteers who joined clinical treatment trials after becoming ill with COVID-19. Connecting with researchers to collect these data in the setting of illness proved challenging.

Finally, from September 2020 to December 2020, when CRVL recruited >50,000 volunteers in <75 days, it was the height of the pandemic and no FDA-approved vaccines for COVID-19 existed. Americans were eager to put an end to the pandemic; it is possible the rapid recruitment success of CRVL could be attributable to these unique urgent conditions.

## Conclusions

Tailored strategic multimedia outreach can successfully recruit women and individuals of diverse racial and ethnic backgrounds into a health research registry. A volunteer registry with diverse participants can assist researchers in enrolling people from under-represented groups for studies. Tailored campaigns designed to recruit specific target audiences for recruitment into health research registries must design messaging and materials based on market research, engagement from stakeholders who reflect and engage with the target audience, and targeted evidence-based communications.

Geotargeted e-mail campaigns sent to Veterans living within the catchment area of VAMCs were found to be a highly effective recruitment tool for clinical trials. E-mail campaigns should be approached in the same way as development of marketing materials, with a clear call to action, easy to understand descriptions of studies, information on study eligibility and how to enroll, and what participation in the study means.

## Supplementary Material

Supplemental data

Supplemental data

## Abbreviations Used

CRVL: Coronavirus Research Volunteer List
PSA: public service announcement
RECOVER: Researching COVID-19 to Enhance Recovery
VA: Veterans Affairs
VAMCs: VA Medical Centers
