# Supplementary material for: Best Practices in Recruitment and Outreach to Women and Diverse Veterans for Coronavirus Research at the U.S. Department of Veterans Affairs
Source: Health Equity. 2023 May 26;7(1):351–5. doi: 10.1089/heq.2023.0013 (PMC10259601; doi:10.1089/heq.2023.0013)
Supplement: Supplemental data [file Suppl_FileS1.pdf]

Coronavirus research at VA

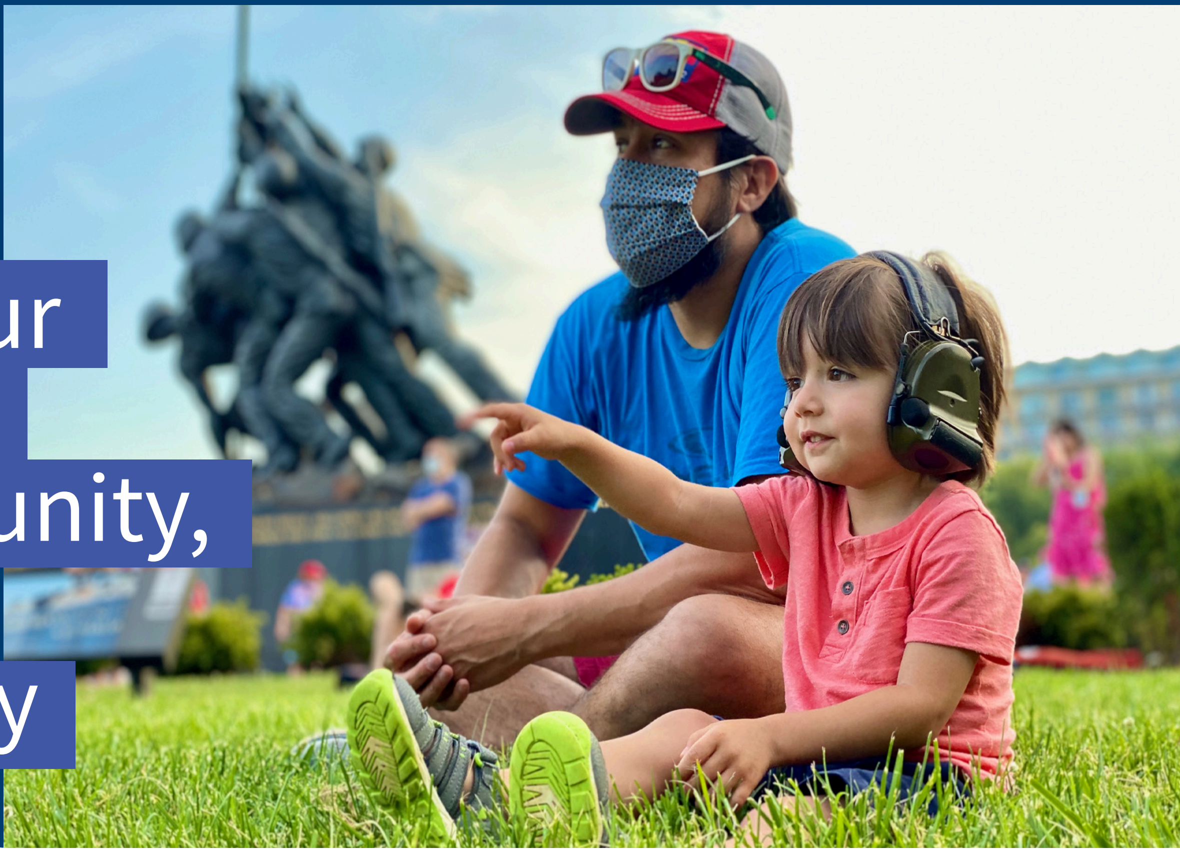

For your family, community, and country

How can I help?

As one of the nation's leaders in health research, we're working to find ways to prevent and treat the coronavirus disease (COVID-19). We're recruiting participants for vaccine clinical trials and other COVID-19 research studies now.

Who can participate?

Anyone over age 18 can sign up, including Veterans, friends and family of Veterans, and VA staff.

How can I sign up?

Go to **www.va.gov/coronavirus-research** to learn more and volunteer today.

VA | U.S. Department of Veterans Affairs
