# Supplementary material for: Best Practices in Recruitment and Outreach to Women and Diverse Veterans for Coronavirus Research at the U.S. Department of Veterans Affairs
Source: Health Equity. 2023 May 26;7(1):351–5. doi: 10.1089/heq.2023.0013 (PMC10259601; doi:10.1089/heq.2023.0013)
Supplement: Supplemental data [file Suppl_FileS2.pdf]

Coronavirus research at VA

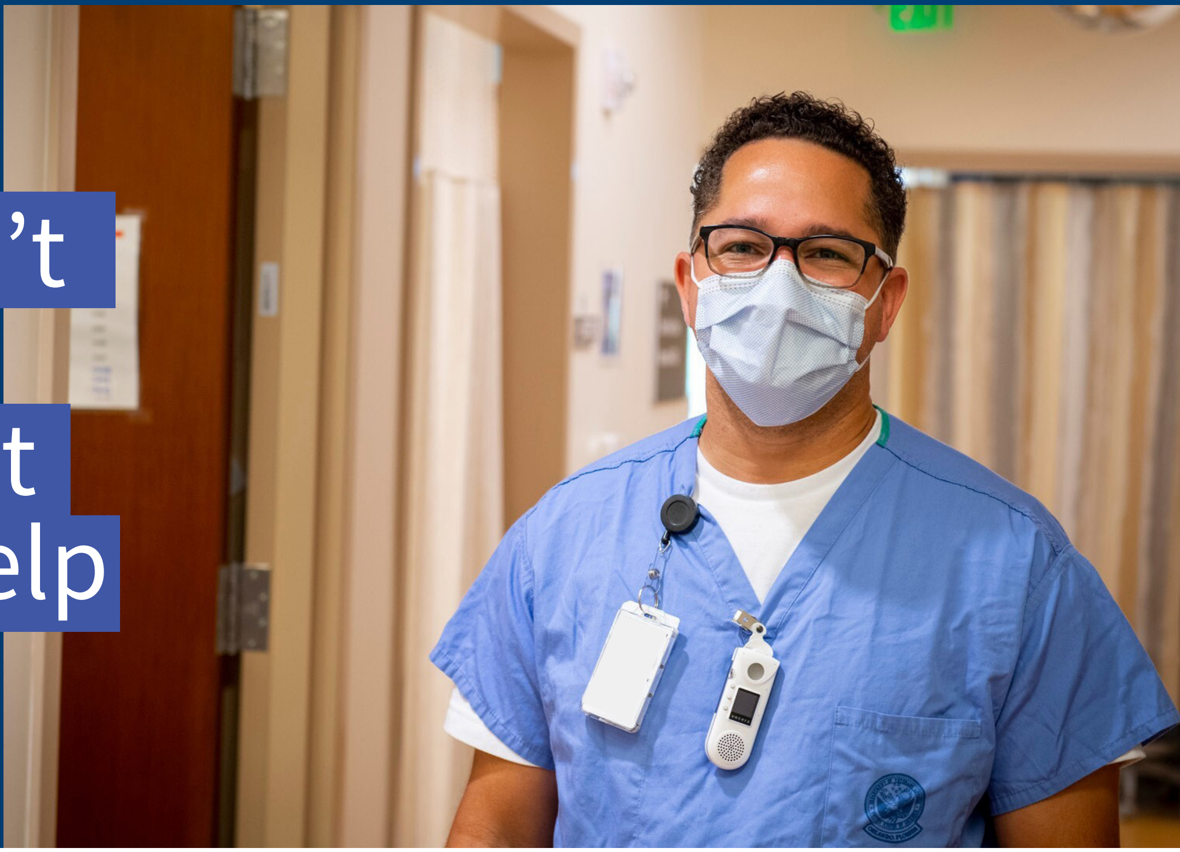

We can’t do it without your help

How can frontline staff help?

Patient referrals and **word-of-mouth** are key to clinical trial recruitment and success. This is more important than ever as VA conducts a number of timely COVID-19 research studies, including several vaccine clinical trials through Operation Warp Speed. Help us spread the word, and invite Veterans to participate in these trials by signing up for VA’s coronavirus research volunteer list.

Who can participate?

Anyone over age 18 can sign up, including Veterans, friends and family of Veterans, and VA staff.

How can patients and staff sign up?

Go to
www.va.gov/coronavirus-research
to learn more and volunteer today.

VA | U.S. Department of Veterans Affairs
